# Sediment structure at the equatorial mid-atlantic ridge constrained by seafloor admittance using data from the PI-LAB experiment

**DOI:** 10.1007/s11001-020-09402-0

**Published:** 2020-02-15

**Authors:** Utpal Saikia, Catherine Rychert, Nicholas Harmon, J. M. Kendall

**Affiliations:** 1 Ocean and Earth Science, University of Southampton, Southampton University, Southampton, UK; 2 School of Earth Sciences, University of Bristol, Bristol, UK

**Keywords:** Ocean bottom seismometers, Admittance function, Sediment properties, Shear wave velocity

## Abstract

Well-constrained marine sediment characteristics (sediment thickness and shear wave velocity) are important not only for the study of climate over geologic times scales but also for correcting and accounting for its presence in seismic data used to investigate deeper structures. We use data from the PI-LAB (Passive Imaging of the Lithosphere Asthenosphere Boundary) experiment, which consisted of 39 broadband ocean bottom seismometers deployed at the Equatorial Mid-Atlantic Ridge near the Chain fracture zone covering 0–80 Myr old seafloor. We compute admittance between the pressure to the vertical displacement at the seafloor at frequencies between 0.1 and 0.2 Hz for microseism-generated Rayleigh waves for 18 stations where data quality is good to determine the sediment thickness and shear wave velocity. We find a general trend of increasing sediment thickness with the seafloor ages, as expected with sediment thicknesses that range from 10–450 m and, shear wave velocities that range from 0.05–0.34 km/s. We find sediment thickness varies almost uniformly across both sides of the ridge, and it indicates that both sides experienced a similar sedimentation process. Our results are in good agreement with the global sediment model that is based on drilling cores and active source experiments, but thinner by up to 50 m at several stations on seafloor older than 25 My. Overlap of the 95% confidence regions between admittance and Ps estimates for thickness and shear velocity is found at 15 stations where we have both Ps and admittance estimates. It suggests that both methods yield accurate estimates for sediment thickness. In addition, our admittance result extends the lateral resolution of sediment characteristics to stations that were not previously resolved by Ps.

## Introduction

Broadband Ocean Bottom Seismometers (OBS) are deployed on the seafloor often coupled to the solid earth on marine sediments. These deployments provide an opportunity to estimate the properties of the marine sediments, such as their average thickness and seismic velocities using a variety of techniques using both body (Harmon et al. 2007; Agius et al. 2018; Rychert et al. 2018) and surface waves (Ruan et al. 2014; Lewis et al. 1998; Bell et al. 2015). These estimates are useful to enhance the further analysis of seismic phases (Harmon et al. 2007) to prevent mapping seismic delays associated with sediments into deeper structure and can also provide information about the sedimentation rates which are important for climate estimates over geological timescales (Agius et al. 2018). The passive seismic methods also complement traditional methods used to determine the shear and compressional properties, including for instance, active source seismic or drill core analyses (Dorman and Jacbson 1981; Berge et al. 1991; Essen et al. 1998).

In this paper, we use the spectral ratio between the pressure and vertical component of the OBS for microseism-generated Rayleigh waves propagating past the station to estimate sediment properties beneath the seismic station. This method differs from the compliance method, which also uses the spectral ratio of the pressure and vertical components, but at lower frequencies, due to loading by ocean infragravity waves at lower frequencies ~ 0.001–0.02 Hz (Crawford et al. 1991). We present results from the Passive Imaging of the Lithosphere-Asthenosphere Boundary (PI-LAB) experiment deployed on and around the equatorial Mid Atlantic Ridge (Harmon et al. 2018; Agius et al. 2018). We compare our results to estimates for sediment properties using Ps converted waves from the same data set and the global compilation of sediment thickness (Straume et al. 2019) and then examine the trend with the age of the seafloor or sedimentation rate and also the velocity-depth relationship of the sediment.

## Data and methodology

**Fig. 1:**
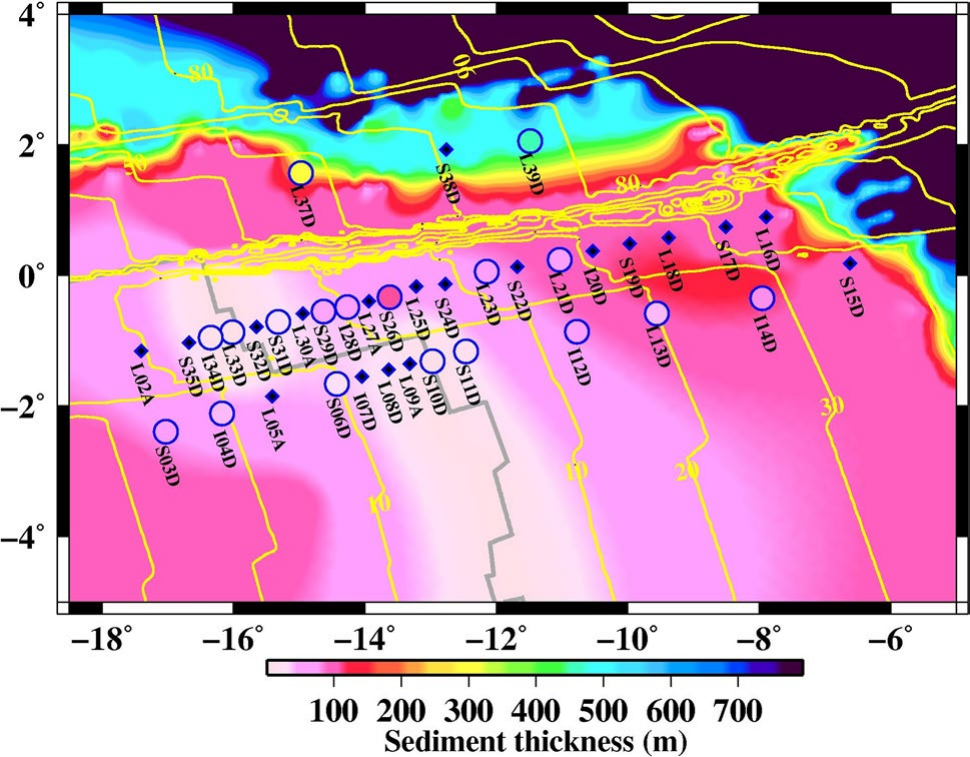
Comparison of estimated sediment thickness with the global sediment thickness model. Seafloor age is shown as yellow contours (Muller et al. 2008). Estimated sediment thickness (colours within blue circles) is overlaid on the global sediment thickness model (background colour). The filled blue color diamonds show the seismic locations where we not able to measure sediment thickness. Grey line shows the location of the Mid-Atlantic Ridge plate boundary

In the present study, we use data from the PI-LAB experiment, which includes 39, 3-component broadband Ocean Bottom Seismometers (OBS) each equipped with a differential pressure gauge (DPG), deployed from March 2016 to March 2017 (Fig. 1). We use the vertical component and DPG data for this study. Although, initially 39 stations were installed, 2 (I01D and I36D) were not recovered, 15 had technical errors caused by lack of recording of one or more channels, contamination from frequent leveling, flooded sensor and/or short duration of recording (weeks to a few months). The remaining 22 stations recorded data on all four components for at least 11 months, four of which still had low coherence (< 0.8) either owing to noise or higher mode contamination (Table 2). We preprocess the data by down sampling to 1 Hz, removing the instrument response between 0.005 and 0.5 Hz. The tilt noise is removed from the vertical component before calculating the pressure-to-vertical displacement transfer function (Crawford and Webb 2000).

Seafloor admittance can be described by the complex transfer function n(ω) in frequency ω between the vertical displacement u_z_(ω) and the differential pressure ∆P(ω) at the seafloor (Ruan et al. 2014; Bell et al. 2015).

1 $${\text{n}}({\upomega }) = \frac{{{\text{u}}_{{\text{z}}} \left( {\upomega } \right)}}{{\Delta {\text{P}}\left( {\upomega } \right)}}$$

The vertical displacement and normal stress for the predicted admittance function was estimated based on the surface wave dispersion code from Computational Programs in Seismology (Herrmann 1978) at frequencies from 0.02 to 0.2 Hz.

To avoid bias in the presence of noise, the transfer function is calculated from the following formula for each day long record of the data:

2 $${\text{n}}\left( {\upomega } \right) = \frac{{ < {\text{u}}_{{\text{z}}} \left( {\upomega } \right) \cdot \Delta {\text{P}}*({\upomega }) > }}{{ < \Delta {\text{P}}({\upomega }) \cdot \Delta {\text{P}}*({\upomega }) > }}$$

where the angle brackets indicate averaging over a number of individual time windows or samples, and the asterisk indicates the complex conjugate. We use a moving 2048s time window with 50% overlap to evaluate (2) using Welch’s method. We determine the coherence γ 2 (ω) of n(ω) using the same Welch’s method:

3 $$\gamma^{2} \left( \omega \right) = \frac{{\left\lceil {\left\langle {u_{z} \left( \omega \right) \cdot \Delta P*(\omega )} \right\rangle } \right\rceil^{2} }}{{\left\langle {u_{z} \left( \omega \right) \cdot u_{z} *(\omega )} \right\rangle \left\langle {\Delta P\left( \omega \right) \cdot \Delta P*(\omega )} \right\rangle }}$$

Days where the average coherence was > 0.8 between 0.1 and 0.2 Hz were averaged to generate n(ω) used in the study below.

**Fig. 2:**
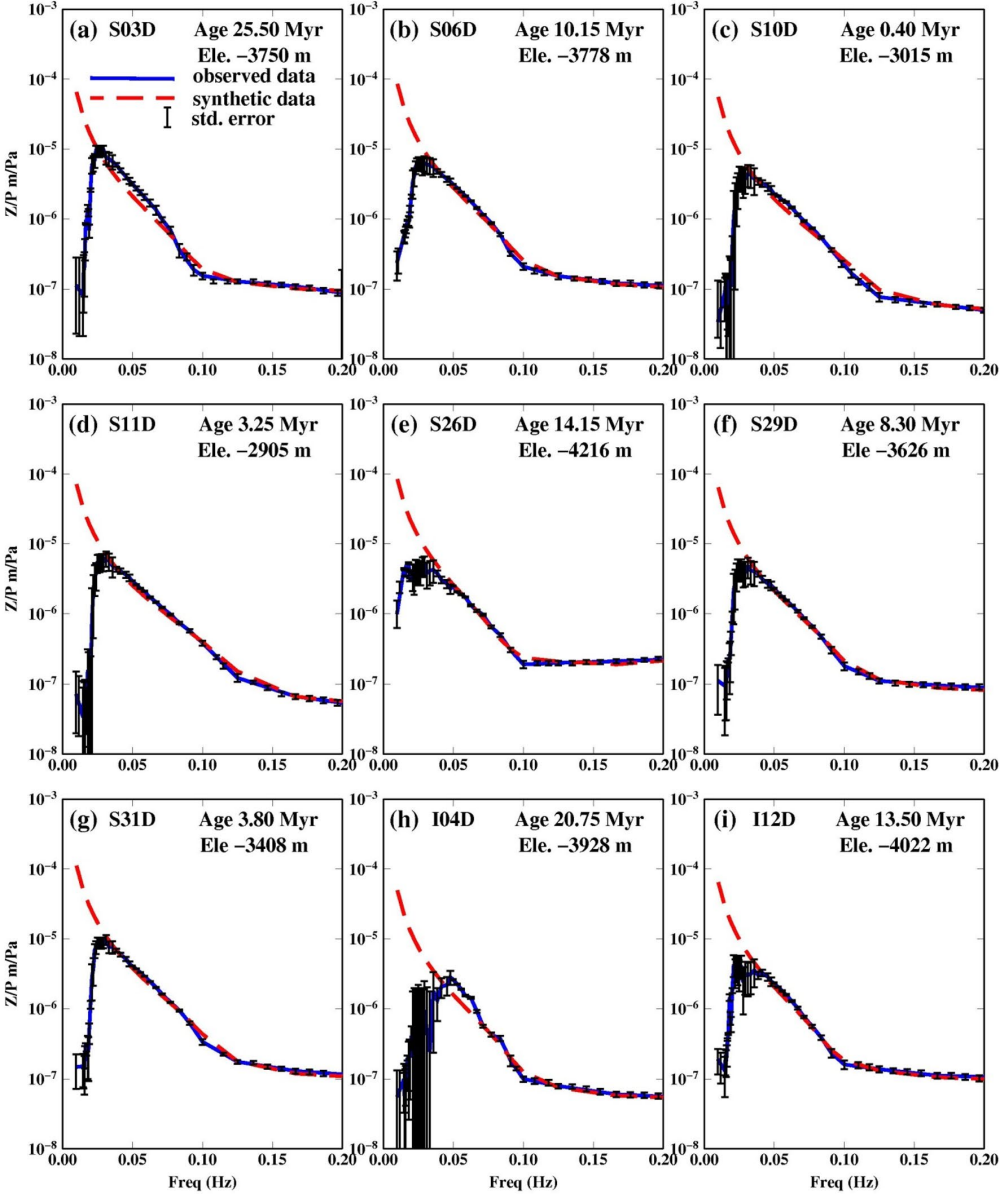

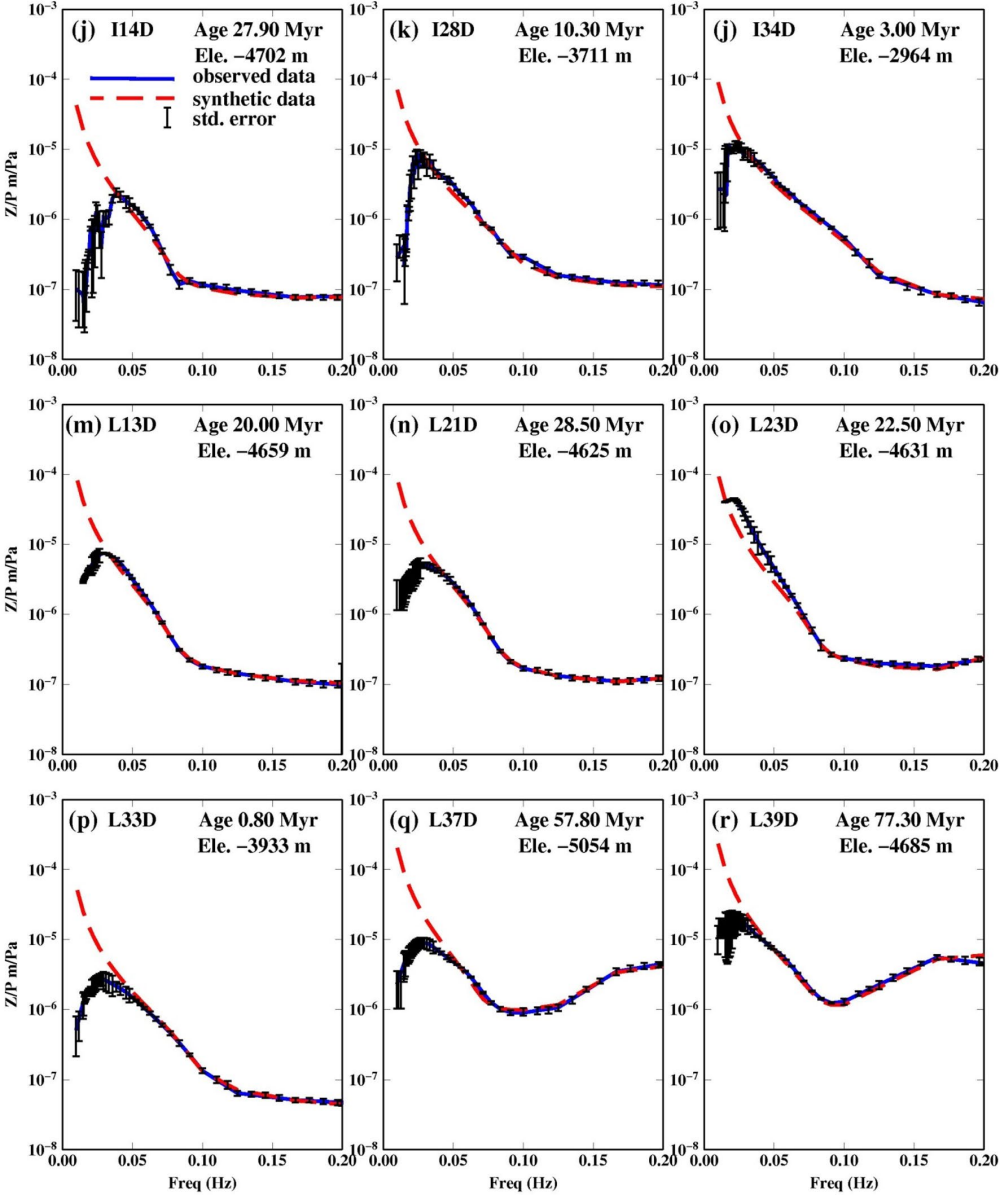
Data fits from inversion results at each station. Water depths and seafloor ages are reported in the top right hand corner. The observed admittance functions are shown as blue lines and the best fit synthetics are shown as red, dashed lines. We only determine sediment properties using frequencies > 0.1 Hz, but show lower frequencies (0.02—0.1 Hz) which are only sensitive to the water column to demonstrate the good calibration achieved with the pre-factor

We invert the transfer function to constrain the sediment structure using a grid search method over sediment thickness and shear velocity. We fit the data in the frequency range between (0.1 and 0.2) Hz where seafloor deformation is dominated by Rayleigh waves (Webb et al. 1991). The grid search method varies sediment thickness ($\mathrm{h})$ with an increment of 0.01 km from 0.01 to 0.40 km thickness and shear wave speed (V_s_) with an increment of 0.02 km/s from 0.01 to 0.4 km/s to minimize the difference between the predicted and observed admittance function between 0.1 and 0.2 Hz. For one station (L39D), where we did not find a satisfactory fit, we increased the grid search area (${\mathrm{v}}_{\mathrm{s}}$ up to 0.5 km/s and $\mathrm{h}$ is up to 0.6 km). We allow for a pre-factor that accounts for imperfect calibration of the pressure records and the vertical relative to the predicted models. The pre-factor represents the shift required to align the synthetic admittance curve to the data in the frequency range between 0.02 and 0.1 Hz where the pressure record is only sensitive to the water column, i.e., avoiding higher frequencies where we are modelling sediment characteristics. The fit result is demonstrated at 0.02–0.1 Hz in Fig. 2. The pre-factor provides the gain correction for the DPG, and is equivalent to the result of approaches that have used pressure-to-acceleration ratios (Zha and Webb 2016) as has been noted previously for admittance studies (Ruan et al. 2014). The pre-factor is the DPG gain factor assuming there are no gain issues with the seismometer. The water depth at each station is fixed to observed value from the multibeam bathymetry collected during the seismic deployment.

We use a seismic velocity model for the crust and uppermost mantle obtained from Ruan et al. (2014) with minor modifications made in-terms of layer thickness and velocities (Table 1). P-wave velocity in the sediment layer is fixed at 1.75 km/s, and density is set to 2000 kg/m^3^, although testing with a range of other values (1.65 km/s and 1.85 km/s) indicated that they have little effect on the final result. To study the effect of initial crustal and upper most mantle velocity on the inversion results, we also performed inversion using Crust 1.0 (Laske et al. 2013) and the velocity model derived from surface wave analysis of the study region (Rychert et al. 2017). Testing with these other velocity models indicated they have little effect on the final results.

**Table 1:** Assumes model parameters, altered from Ruan et al. (2014)

Layer	Thickness(km)	$\rho$ (g/cm^3^)	${v}_{p}$(km/s)	${v}_{s}$(km/s)
Water	Local, fixed	1.03	1.53	0.0
Sediment	Variable	2.00	1.75	–
Upper crust	3	2.40	5.10	2.65
Lower crust	6	3.15	6.90	3.95
Uppermost mantle	20	4.30	7.90	3.35

For each OBS, the 95% confidence region is determined by a grid search and defined by the following equation (Draper and Smith 1998).

4 $$S(\theta ) = S(\hat{\theta })\left[ {1 + \frac{p}{n - p}F^{\prime}\left( {p,n - p,1 - \alpha } \right)} \right]$$

where $S\left( {\hat{\theta }} \right)$ defines the best fit variance, $\mathrm{p}$ is the number of parameters (p = 2), $\mathrm{n}$ is the number of observations (n = 11, the number of frequencies between 0.1 and 0.2 Hz), and ${\alpha }$ defines the confidence of 95% interval. $F^{\prime}(p,n - p,1 - \alpha )$ is the F distribution. We note that in calculating our confidence regions our choice of the number of observations is conservative, and could possibly be smaller than previous studies (Bell et al. 2015; Ruan et al. 2014). For instance, we could have chosen the number of observations as the number of frequencies multiplied by the number of admittance curves averaged in the stack.

To check the conformity, we compare the present result with the global sediment thickness (Straume et al. 2019) and earlier published result using P-to-s (Ps) conversions (Agius et al. 2018). We checked whether present result overlaps the 95% confidence region of the Ps result using previously reported dt and 1σ error bars (Agius et al. 2018), and the following equation to estimate sediment thickness:

5 $$dt = h\left( {\sqrt {\left( {{1 \mathord{\left/ {\vphantom {1 {V_{s} }}} \right. \kern-\nulldelimiterspace} {V_{s} }}} \right)^{2} - u^{2} } - \sqrt {\left( {{\raise0.7ex\hbox{$1$} \!\mathord{\left/ {\vphantom {1 {V_{p} }}}\right.\kern-\nulldelimiterspace} \!\lower0.7ex\hbox{${V_{p} }$}}} \right)^{2} - u^{2} } } \right)$$

where $\mathrm{d}\mathrm{t}$, $\mathrm{h}$, $\mathrm{u}$, ${\mathrm{v}}_{\mathrm{p}}$ and ${\mathrm{v}}_{\mathrm{s}}$ represent the delay time, sediment thickness, horizontal slowness, P- and S-wave velocity, respectively. We assumed an average slowness ($\mathrm{u}$) value of 0.60 s/km and an average ${\mathrm{v}}_{\mathrm{p}}$ value of 1.75 km/s, and varied shear wave velocity from 0.01 to 0.4 km/s with an increment of 0.01 km/s.

## Results

**Fig. 3:**
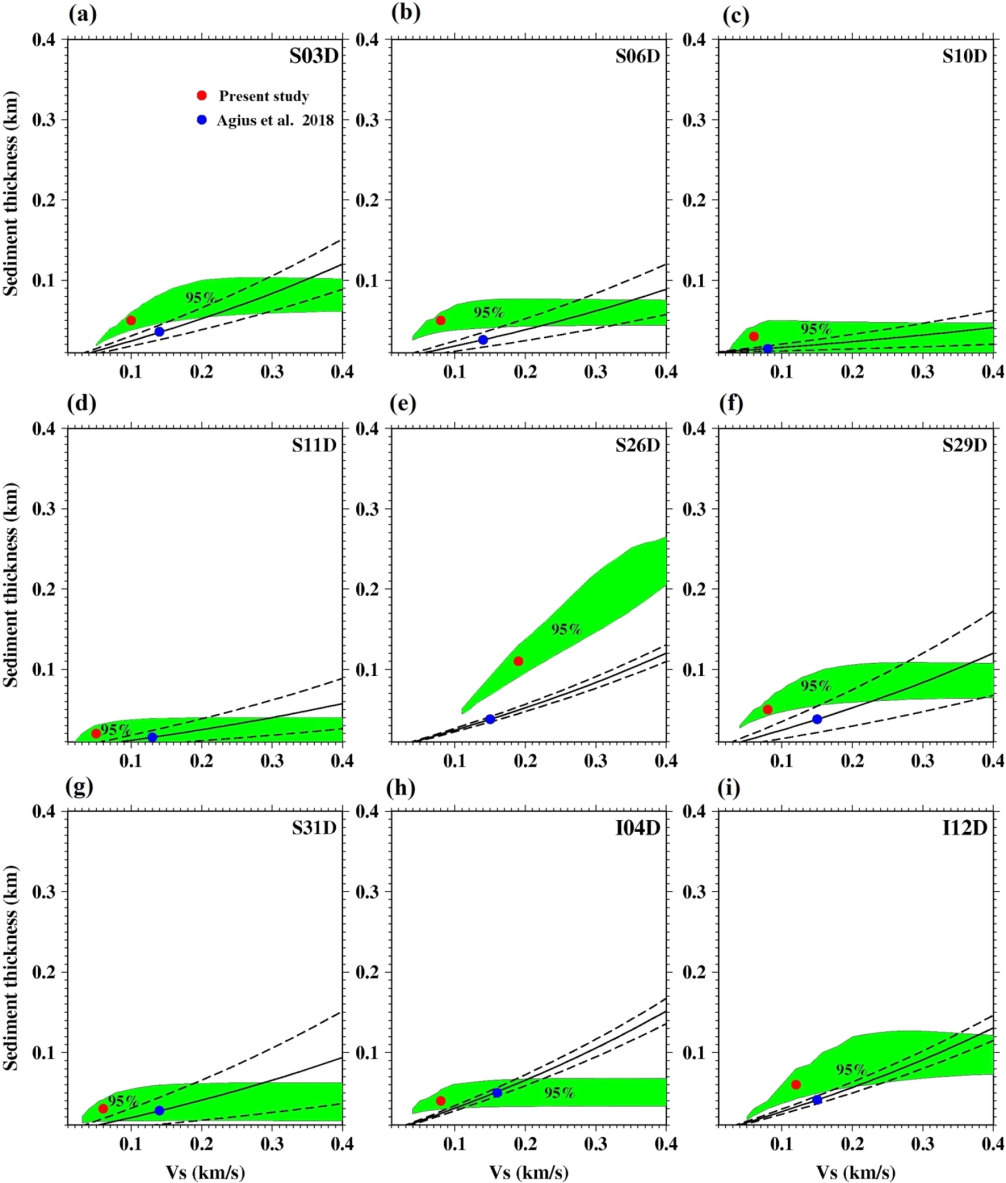

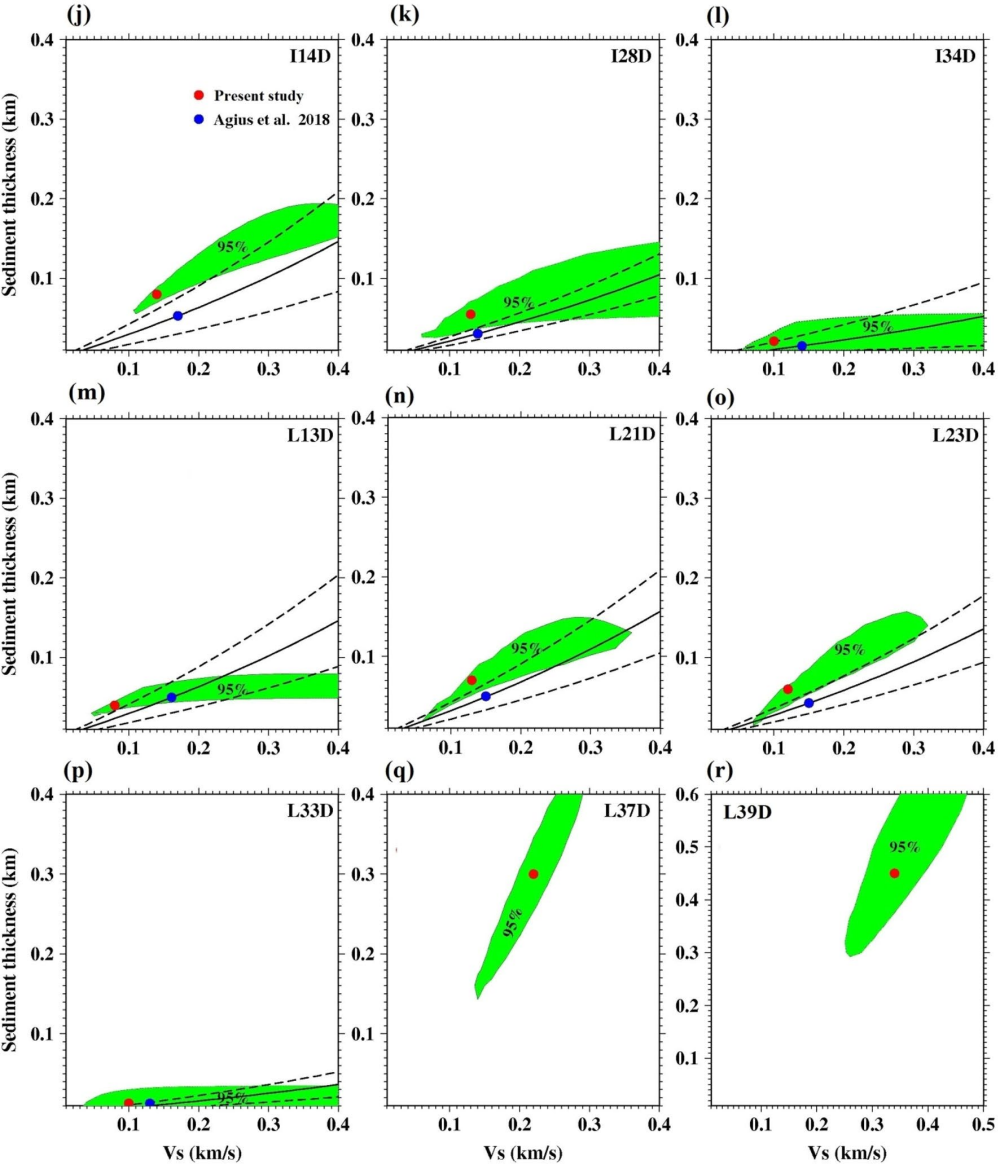
Grid search results for best fitting sediment thickness and shear wave velocity for each stations. Green area shows the 95% confidence region of our study, while red circle indicates the optimum value obtained from admittance data. The blue circle indicates the optimum value obtained from the Ps study (Agius et al. 2018). The two dashed line indicate the region of acceptable value from Ps delay time with 1$\sigma$ error bounds assuming a v_p_ of 1.75 km/s

**Fig. 4:**
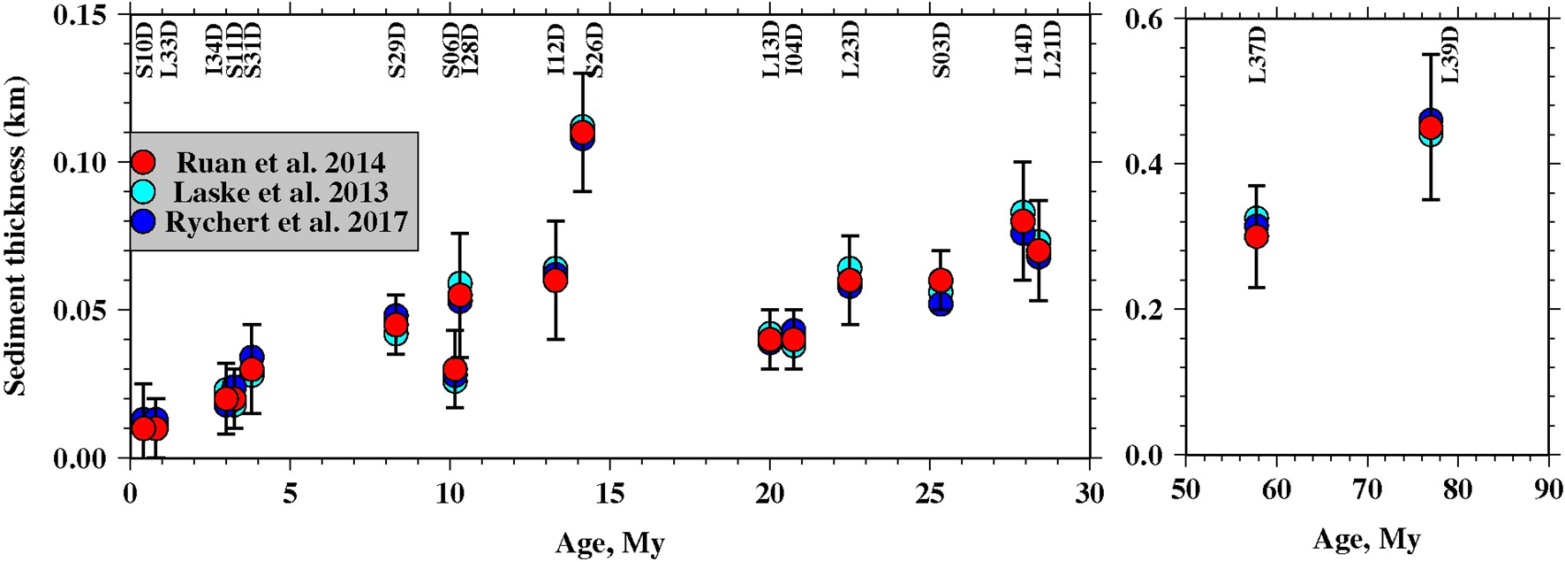
Effect of sediment thickness using different velocity models. Red circles define the values obtained from the model of Ruan et al. (2014), while cyan and blue circles show the value obtained from Crust.1 (Laske et al. 2013) model and the velocity model of Rychert et al. (2017), respectively

We report results for 18 stations with good coherence (> 0.8) (Table 2). The admittance function pattern varies as it moves away from the ridge, becoming more U-shaped and concave upwards as the seafloor age increases (Fig. 2). The fit to the data for the selected 18 stations is shown in Fig. 2. The average estimated sediment thickness, shear wave velocity and corresponding error of the 18 stations are listed in Table 2 as well as the DPG gain factors determined from the grid search. The best fit from the grid search results are shown in Fig. 3 along with the 95% confidence region. The error surfaces of stations S26D, L37D and L39D have a different trend than the rest of the stations, as expected, reflecting the larger trade-off between velocity and thickness in locations with thicker sediment (Fig. 3). The effect of using different crustal velocity models on the inversion results is shown in Fig. 4.

**Table 2:** PI-LAB stations latitude, longitude, elevation, seafloor age, average sediment thickness (h), average shear wave velocity (v_s_$$)$$ and the corresponding error

Station	Long. (^o^)	Lat. (^o^)	Ele. (m)	Age (Myr)	Thick* (h) (m)	Avg.v_s_ (km/s)	Err (h) (m)	Err v_s_ (km/s)	DPG gain
I04D	− 16.1733	− 2.1238	− 3928	20.75	40	0.08	10	0.18	1.22
I07D	− 14.0428	− 0.5565	− 3819	7.80	TE	–	–	–	
I12D	− 10.7766	− 0.8683	− 4022	13.30	60	0.12	20	0.07	1.26
I14D	− 7.9524	− 0.3522	− 4702	27.90	80	0.14	10	0.02	1.31
I20D	− 10.5352	0.3681	− 4724	31.10	TE	–	–	–	
I28D	− 14.2684	− 0.4918	− 3711	10.30	55	0.13	21	0.15	1.54
I34D	− 16.3485	− 0.9579	− 2964	3.00	20	0.10	12	0.16	2.13
L02A	− 17.4085	− 1.1667	− 3499	10.60	TE	–	–	–	
L05A	− 15.4100	− 1.8600	− 4052	15.95	TE	–	–	–	
L08D	− 13.6409	− 1.4493	− 3357	4.25	TE	–	–	–	
L09A	− 13.3185	− 1.3569	− 3378	2.05	TE	–	–	–	
L13D	− 9.5619	− 0.5862	− 4659	20.00	40	0.08	10	0.03	1.71
L16D	− 7.8953	0.8933	− 4581	45.10	TE	–	–	–	–
L18D	− 9.3765	0.5769	− 4890	36.90	LC	–	–	–	–
L21D	− 11.0380	0.2364	− 4625	28.40	70	0.13	17	0.05	1.63
L23D	− 12.1478	0.0521	− 4631	22.50	60	0.12	15	0.01	2.01
L25D	− 13.2230	− 0.1745	− 4207	16.60	TE	–	–	–	–
L27A	− 13.9427	− 0.4007	− 3928	12.00	TE	–	–	–	–
L30A	− 14.9467	− 0.5880	− 4003	6.00	TE	–	–	–	–
L33D	− 16.0152	− 0.8747	− 3933	0.80	10	0.01	10	0.18	1.21
L37D	− 14.9718	1.5657	− 5054	57.80	300	0.22	70	0.02	1.32
L39D	− 11.4904	2.0557	− 4685	77.00	450	0.34	100	0.06	1.24
S03D	− 17.0315	− 2.4021	− 3750	25.35	60	0.10	10	0.03	2.19
S06D	− 14.4298	− 1.6703	− 3778	10.15	30	0.08	13	0.18	2.16
S10D	− 12.9697	− 1.3180	− 3015	0.41	10	0.06	15	0.18	1.39
S11D	− 12.4602	− 1.1691	− 2905	3.25	20	0.05	10	0.19	1.72
S15D	− 6.6228	0.1814	− 4927	35.70	TE	–	–	–	–
S19D	− 9.9754	0.4809	− 4607	34.00	TE	–	–	–	–
S24D	− 12.7806	− 0.1383	− 4453	18.70	TE	–	–	–	–
S22D	− 11.6799	0.1254	− 4352	24.95	LC	–	–	–	–
S24D	− 12.7806	− 0.1383	− 4453	18.70	TE	–	–	–	–
S26D	− 13.6260	− 0.3434	− 4216	14.15	110	0.19	20	0.03	1.85
S29D	− 14.6272	− 0.5597	− 3626	8.30	45	0.08	10	0.04	1.53
S31D	− 15.3187	− 0.7141	− 3408	3.80	30	0.06	15	0.18	2.14
S32D	− 15.6470	− 0.7968	− 2967	1.70	TE	–	–	–	–
S35D	− 16.6798	− 1.0372	− 3773	5.50	TE	–	–	–	–
S38D	− 12.7623	1.9218	− 4926	71.00	LC	–	–	–	–

Stations labeled TE had a technical error caused by either: lack of recording of one or more channels, contamination from frequent leveling, flooded sensor and/or short duration of recording (weeks to a few months). Stations labeled LC represent stations that recorded data on all four components for at least 11 months, but still had low coherence either owing to noise or higher mode contamination. Stations I01D and I36D were never recovered from the ocean floor

**Fig. 5:**
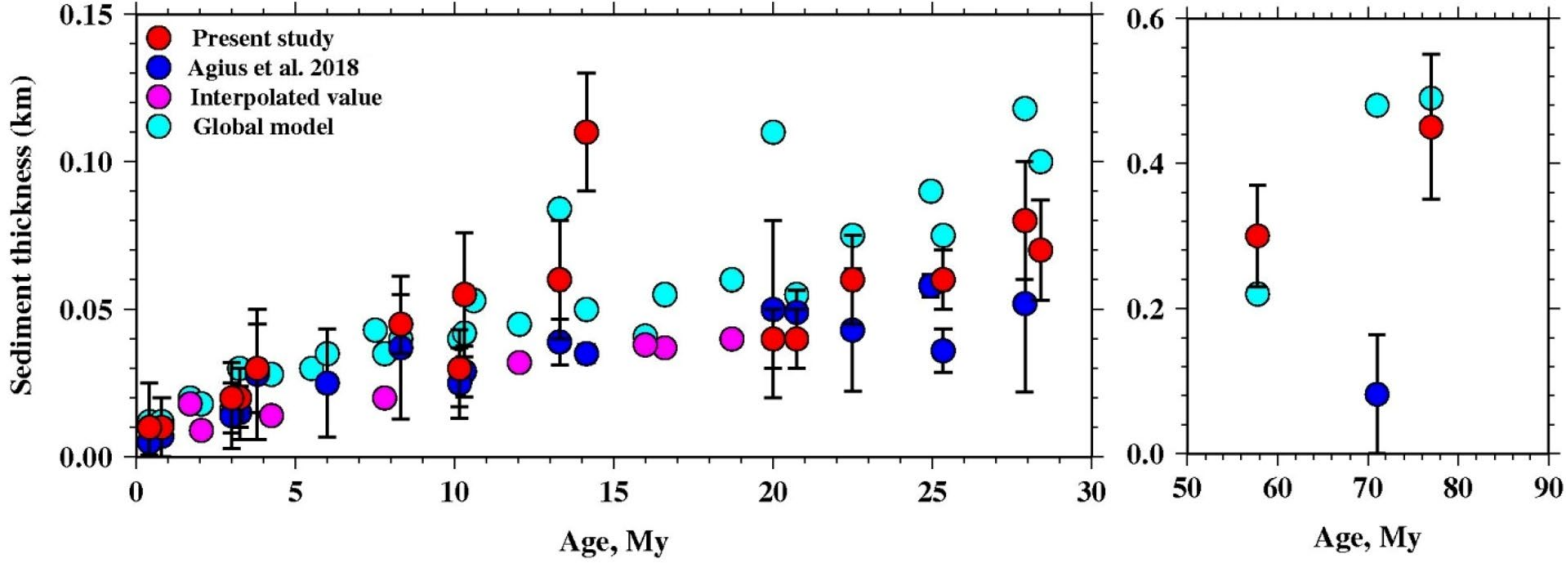
Variation of sediment thickness with seafloor age. Red circles show the results of present study, while cyan and blue circles show the global model and Ps result (Agius et al. 2018), respectively. The magenta circles show the locations where the Ps study did not resolve sediment characteristics, but reported values based on interpolation based on nearby stations. 95% confidence limits are shown by black error bars

We present the inversion results for each station in terms of seafloor ages (Fig. 5). For 0–10 My old seafloor estimated sediment thickness varies from 10 to 45 m and it increases with the seafloor ages (Fig. 4). The maximum thickness is detected at S29D station (seafloor age 8.30 Myr), while the minimum (10 m) is found at stations S10D and L33D (seafloor age 0.41–0.80 Myr). For 10–20 My old seafloor, the maximum sediment thickness is detected at S26D station of about 120 m, thicker than the surrounding stations, which range from 30–60 m (Fig. 4). For 20–40 Myr old seafloor, the inferred sediment thickness is 40–80 m (Fig. 4). For 41–77.5 Myr old seafloor we find thicknesses of 300–450 m.

## Discussion

Comparison of the 95% confidence regions of the admittance study presented here and the 1$\upsigma$ confidence regions of the Ps converted phase study of Agius et al. (2018) is shown in Fig. 3. The confidence regions overlap in 15 out of 16 stations where both techniques were used, even if the best fitting parameters estimated between the two studies do not necessarily lie within each other’s confidence limits. Typically, our best-fitting thickness is similar to that from Ps, but our velocity result is slower. However, the Ps study assumed the velocity-thickness relationship determined by Ruan et al. (2014) for Cascadia. This could suggest that sediment properties in the Atlantic are not the same as Cascadia. Both methods have good sensitivity to the sediment thickness at low thickness (< ~ 200 m), but relatively poor sensitivity to shear velocity, evidenced by the elongation of the confidence regions along the shear velocity axis. The two confidence regions did not overlap in one case, site S26D, where the admittance method indicated 110 m sediment thickness and the Ps delay indicated 35–50 m (Aguis et al. 2018). In this region, it may be that the Ps phase is sensitive to layering within the sediment, thus giving an artificially thin result. In other studies, Ps has been shown to be consistent with estimates from compliance studies in thicker sediments up to 1.5 km in the Pacific Hawaii (Doran and Laske 2019). Consistency between the methods may depend on the structure and type of the sedimentation.

Our trends in sediment thickness with age are generally consistent with the notion that oceanic sediments thicken with increasing age of the lithosphere (Olson et al. 2016; Sclater 2003) (Fig. 5). The estimated sediment thickness varies from 10–20 m at the ridge axis and reaches up-to 400–450 m on the seafloor older than 70 Myr. At > 70 My seafloor the greater thickness likely reflects both the longer sedimentation times and also greater terrigenous sediment input from Africa due to the proximity of the continent and also the prevailing winds (Ruddiman and Janecek 1989). The observed sediment thickness varies almost uniformly across both sides of the ridge and indicates that both sides might have experienced by the same sedimentation process.

We compare our estimated sediment thicknesses as a function of age with the global sediment thickness model (Straume et al. 2019) and an earlier published result (Agius et al. 2018) (Fig. 5). At young ages (< 10 Myr), our sediment thicknesses are within the error of the global model. At two stations S10D and L33D near the ridge our sediment estimate is 10 m, and the error bars overlap 0 km, suggesting that sediment may not be required. From 17–30 My seafloor our sediment thicknesses are about 25–30 m thinner than the global model. At 50–80 Myr seafloor, our sediment thicknesses are similar to the global model. Our sediment thicknesses generally agree with the similar age trend found by the previous Ps study (Agius et al. 2018).

We also compare our result to constraints from the sub-bottom profiler and International Ocean Drilling Project (IODP). Sub-bottom profiler data was only available at a few stations, but at station L13D the two-way travel time was 0.035 s corresponding to a sedimentary thickness of about 54–64 m for ${v}_{p}$ of 1.55–1.84 km/s, in good agreement with our result (40 ± 10 m) (Agius et al. 2018). The IODP cores were drilled on the eastern side of the ridge in seafloor 3.7 Myr old, about 65 and 84 km away from the S11D station. The IODP cores found 200 m sediment thickness in a local sedimentary basin (Ruddiman and Janecek 1989), which is much thicker than most of the stations in the present study and other studies (Aguis et al. 2018; Straume et al. 2019). This is probably explained by the fact that sediment deposition is enhanced by gravity flows and mass wasting in local basins (Ruddiman and Janecek 1989). IODP preferentially samples basins, while we preferentially chose station locations at bathymetric highs, given the advantage for instrument recovery. The larger 110 ± 20 m sediment thickness result at one of our stations, S26D, is likely explained by its location in a flat bottomed local basin. This suggests that even though the sediment is thin across most of the region, there is likely significant local variation by up to ~ 100 m due to local sedimentary processes in addition to pelagic sedimentation.

**Fig. 6:**
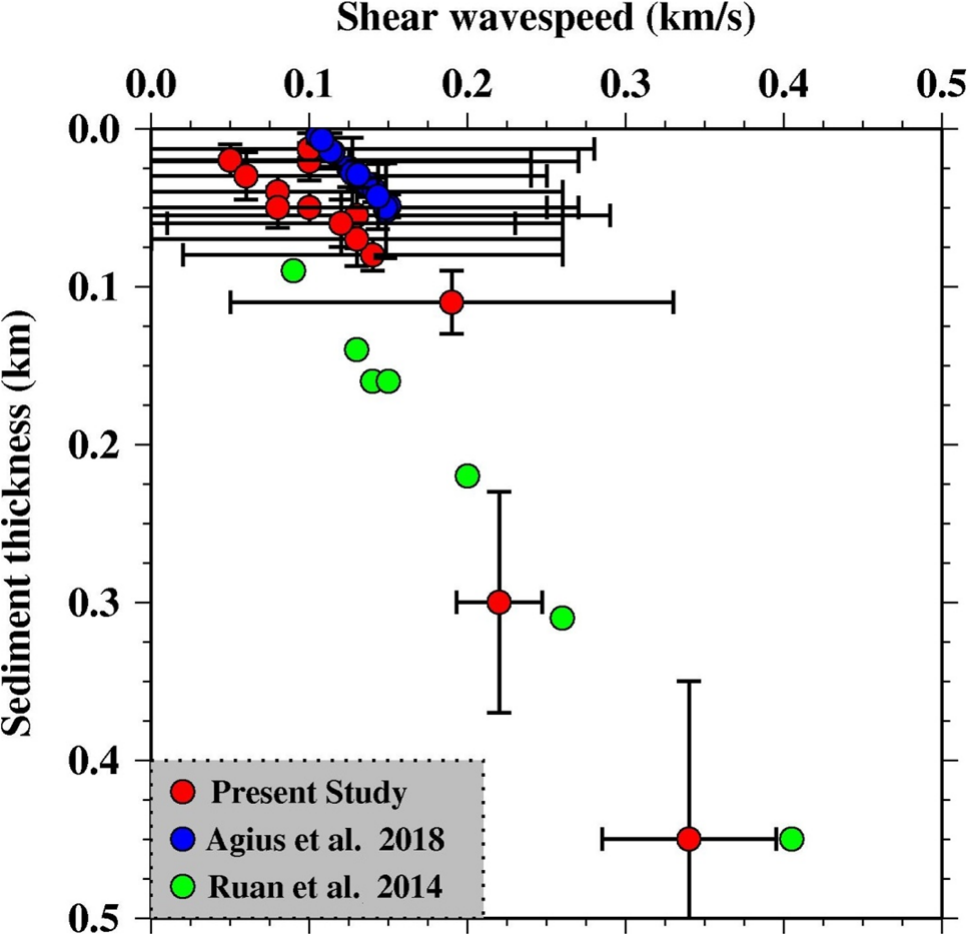
Variation of shear wave velocity with respect to sediment thickness. The red circles show the result of present study. The blue circles show the Ps result from our study area (Agius et al. 2018). The green circles show the Ruan et al. (2014) results from the Juan de Fuca plate. 95% confidence limits are shown as the black error bars

We compare our shear wave speed vs the inferred thickness of the sediment layer to that from Cascadia (Ruan et al. 2014) (Fig. 6). The sediments are much thicker beneath Cascadia than our study region so we couldn’t compare the results from both studies at less than ~ 85 m thickness. At 85–140 m thickness, we have only one measurement which shows higher shear velocity than the Cascadia result but within the error bounds. At sediment thicknesses > 200 m, our sediment thickness and shear velocity estimates are within error of the Cascadia results but slightly slower.

**Fig. 7:**
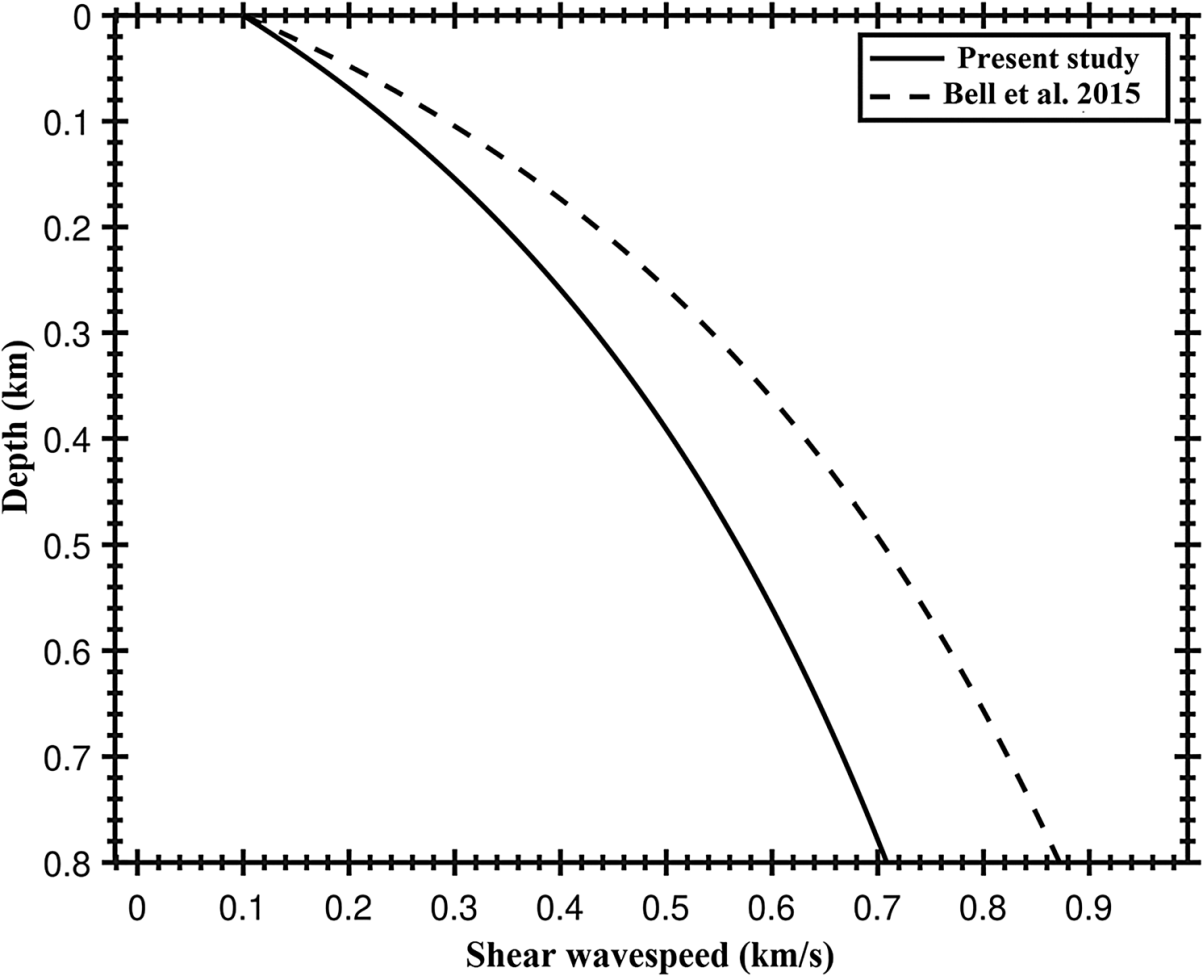
Our sediment velocity-depth relationship (solid) in comparison to that of Bell et al. (2015) (dashed)

We also inverted for a continuous shear velocity, ${\mathrm{v}}_{\mathrm{S}}$, structure as a function of depth after Ruan et al. (2014); Bell et al. (2015) using an equation of the form:

6 $$V_{s} \left( z \right) = \frac{{az^{2} + bz + cV_{0} }}{{\left( {z + c} \right)}}$$

where V_0_ is 100 m/s after Ruan et al. (2014). We solve for coefficients a, b and c using an iterative damped least squares inversion using admittance observations for all stations with an initial estimate of 100 m or more of sediment thickness, assuming the crustal structure given in Table 1. We found that using stations with thinner grid search sediment estimates were not resolvable and destabilized the inversion. We find values for the coefficients of a = 0.09 ± 0.26, b = 1.13 ± 0.22, and c = 0.65 ± 0.20, and our shear velocity function is plotted relative to the Bell et al. (2015) curve in Fig. 7. Our velocity profile is slower than the Bell et al. (2015) curve, although our coefficients are within the error of the Bell et al. (2015) coefficients. Using our new relationship, the sediment thickness estimates for the stations used in the inversion increase to 782 ± 235 m for L37D, 759 ± 225 m for L39D and 222 ± 204 m for S26D, but are within the reported error bounds for the single layer thickness reported in Table 2. The slower velocities could reflect different sediment properties in the Atlantic in comparison to the Pacific.

## Conclusion

We determine the sediment thickness at the Equatorial Mid-Atlantic Ridge using the admittance function obtained for microseism-generated Rayleigh wave. The estimated sediment thickness varies from 10 to 450 m. It is 10–20 m thick at the ridge and it reaches up-to 400–450 m on the seafloor older than 70 Myr. For young seafloor (< 10 My) our estimated thicknesses are consistent with those from a previous study that used Ps conversions and also the global model (Agius et al. 2018; Straume et al. 2019). For the moderate aged lithosphere (17–30 Myr), the best fitting sediment thickness is 25–30 m thinner than the global model, but 5–25 m thicker than the thicknesses based on Ps delay times. For the oldest ages (50—80 My) sediment thickness are similar to the global model. Overlap of the 95% confidence regions between the admittance function and Ps estimates for thickness and shear velocity is found at 15 out of 16 stations where both methods yielded results. It suggests that both methods determine accurate estimates for sediment thickness. In addition, using admittance can expand the numbers of resolved station, in particular in locations where Ps phases could not determine sediment structure.
